# Lectures on Medical Education, or on the Proper Method of Studying Medicine

**Published:** 1864-05

**Authors:** 


### Book Notices.

Lectures on Medical Education, or on the Proper Method of Studying Medicine. By Samuel Chew, M.D., Prof. of Practice and Principles of Medicine and of Clinical Medicine, in the University of Maryland. Philadelphia: Lindsay & Blakiston. 1864.

This is a neat duodecimo volume of 152 pages. It embraces five lectures on the following subjects:—

*Lecture I.* Introduction—Power of Industry—Whether special Talents for Medicine are necessary—What Talents are requisite—Good Senses—Good Sense—Means for the Study of Medicine.

*Lecture II.* Reading as a Means of Study—Errors in its Use, &c., &c.

*Lecture III.* Errors in Reading Continued—Reading without Thinking—Lectures as a Help in the Study of Medicine—Their Utility, &c., &c.

*Lecture IV.* Clinical Experience—Necessity of a Hospital to every Medical School—Conversation as a Means of Acquiring Knowledge, &c., &c.

*Lecture V.* Medical Schools—Controversy Respecting them, &c., &c.

It is a pleasant readable book, containing very many useful suggestions to students; and may be read with profit by all who wish to improve their knowledge of medical science and practice. For sale by S. C. Griggs & Co., Lake street, Chicago.

